# Relationship Between *Helicobacter pylori* IgG Seroprevalence and the Immune Response to Poliovirus Vaccine Among School-Age Children From a Population With Near-Universal Immunity Level

**DOI:** 10.3389/fmed.2021.797719

**Published:** 2022-01-20

**Authors:** Layaly Badran Abu Zher, Merav Weil, Eias Kassem, Nael Elias, Myron M. Levine, Khitam Muhsen

**Affiliations:** ^1^Sackler Faculty of Medicine, Department of Epidemiology and Preventive Medicine, School of Public Health, Tel Aviv University Ramat Aviv, Tel Aviv, Israel; ^2^Central Virology Laboratory, Ministry of Health, Tel Hashomer, Israel; ^3^Department of Pediatrics, Hillel Yaffe Medical Center, Hadera, Israel; ^4^Saint Vincent de Paul-French Hospital, Nazareth, Israel; ^5^Center for Vaccine Development and Global Health, University of Maryland School of Medicine, Baltimore, MD, United States

**Keywords:** poliovirus vaccine, neutralizing antibodies, *H. pylori*, cytotoxin-associated gene A, pepsinogen

## Abstract

**Objectives:**

To examine the association between *Helicobacter pylori* seroprevalence and serum pepsinogens (PGs) as markers of gastric inflammation), with high neutralizing antibody titers to poliovirus type 1 and 3 vaccine strains among children age 3–4 years, subsequent to sub-clinical infection acquired during a wild-type poliovirus type 1 outbreak in Israel.

**Methods:**

A serosurvey was conducted among 336 children aged 5–17 years who were vaccinated with both inactivated polio vaccine and oral polio vaccines. *H. pylori* serum IgG antibodies and PG concentrations were measured using ELISA. Neutralizing antibodies to poliovirus vaccine strains were measured and children with a titer ≥1:8 were considered immune. High-level immunity was defined as having a serum NA titer >1:2048. Propensity score inverse weighting was used to account for confounders.

**Results:**

Neutralizing antibodies titers ≥1:8 to poliovirus type 1 and 3 vaccine strains were found in 99.4 and 98.2% of the children, respectively. An inverse association was found between *H. pylori* seropositivity accompanied by PGI:PGII ratio ≤6.5 (marker of gastric inflammation) and high-level immunity to poliovirus type 1: OR 0.39 (95% CI 0.68–0.91), *p* = 0.027. The association between *H. pylori* seropositivity of CagA virulent phenotype and polio high immunity was not significant. The association between *H. pylori* seropositivity and high neutralizing antibodies to type 3 poliovirus was of low magnitude and not significant.

**Conclusions:**

*H. pylori* seroprevalence accompanied by evidence of gastric inflammation was inversely correlated with high titers of neutralizing antibodies to poliovirus in children from a population with near universal polio immunity.

## Introduction

Polioviruses comprise 3 serotypes that multiply in the human intestine; in a minority of infected persons these can cause paralytic disease, poliomyelitis (1). Since the World Health Assembly adopted a resolution for the worldwide eradication of polio in 1988, the number of polio cases worldwide has declined by nearly 99.9%. Both wild poliovirus type 2 (WPV2) and WPV3 have officially been certified as globally eradicated (no cases of WPV2 have been documented since 1999, and none of WPV3 since 2012). Only two countries (Afghanistan and Pakistan) are currently affected by WPV1 (2, 3). The remarkable progress toward polio eradication is attributed to successful childhood vaccination with the oral polio vaccine (OPV) and the injectable inactivated polio vaccine (IPV) (4).

Immunization with OPV is simple and practical; it generates both systemic humoral (serum antibodies) and intestinal (secretory IgA) immunity. Vaccination with OPV induces superior intestinal immunity than IPV; thus, it can prevent the transmission of wild type viruses. Despite these advantages, OPV poses a risk, albeit very low, of vaccine-associated paralytic poliomyelitis (VAPP) (4). IPV is highly safe and induces high titers of serum antibodies (4). After the certification of WPV2 eradication, a global switch from trivalent OPV (tOPV, containing vaccine virus types 1, 2, and 3) to bivalent OPV (bOPV, containing types 1 and 3) was completed in 2016 (3, 4). At least two doses of IPV in routine immunization are also recommended in countries that vaccinate with OPV only (4, 5) to reduce the risk of VAPP. Presently, polio vaccination is based mainly on OPV (with one dose of IPV) in most low-middle income countries, while IPV is mainly used in high-income countries (4).

The threat remains of poliomyelitis re-emerging from the final global foci to spread elsewhere. This is evidenced by the silent circulation of WPV1 in 2013–2014 in the southern region of Israel, which was identified through environmental surveillance. No clinical cases of paralytic poliomyelitis were detected and molecular analysis found the virus to be similar to viruses circulating in Pakistan and from sewage samples in Egypt (6, 7). This alarming event occurred on the background of using only IPV vaccination during 2005–2013, and consistently high national coverage of polio vaccines in Israel. The silent poliovirus reintroduction, which lasted over 1 year in Israel (2013–2014), was successfully interrupted consequent to major interventions implemented by the Israeli Ministry of Health. These included mass administration of bOPV to stimulate intestinal immunity in all birth cohorts of children under age 9 years, the birth cohorts that were subject to the IPV-only vaccination program implemented during 2005–2013. In addition, OPV vaccination supplementary to IPV was reintroduced at ages 6 and 18 months, as the updated polio immunization policy in Israel (6, 8).

Oral enteric vaccines have demonstrated lower immunogenicity and efficacy in persons living in low-middle income countries, in contrast to persons from high-income countries (9). This was first reported in the 1960s and 1970s, in publications that showed OPV to be markedly less immunogenic in infants in India and other developing countries (10–13). This phenomenon was partly explained by the presence of the immunodominant attenuated type 2 poliovirus in the vaccine, which negatively affected the immune response to types 1 and 3 poliovirus in the vaccine, even as it elicited strong type 2 seroconversions (13); and to low vaccine take due to concurrent enteric infections at the time of vaccination (11, 14). An association was found of diminished immunogenicity and belonging to low socioeconomic status communities (14). Moreover, significantly lower seroconversion rates were found for poliovirus types 1, 2, and 3 following immunization with IPV in infants from Puerto Rico, compared to US infants (15). This suggested differences between the two populations, in the immune response to injectable poliovirus vaccines, as well.

*Helicobacter pylori*, a bacterium that persistently colonizes the stomach, is acquired in early childhood and causes asymptomatic gastritis; in a minority of infected persons peptic ulcers develop (16), and gastric cancer usually in later adulthood (17). Cytotoxin-associated gene A (CagA) antigen is main virulence factor of *H. pylori*. The cag pathogenicity island of *H. pylori* encodes for a type-IV secretory apparatus through which CagA antigen is inserted into the host cell [reviewed by Surbaum and Michetti (18)]. Infection with *H. pylori* CagA positive strains was linked with increased risk for peptic ulcers, premalignant gastric lesions and gastric cancer (17, 19). *H. pylori* have additional antigens such as VacA, Omp and NapA and others, however only a few of these antigens showed positive associations with gastric cancer (20–22). Additionally, the association between CagA sero-positivity and gastric cancer was of greater magnitude than other antigens (20, 21). Following adjustment for the presence of other antigens, CagA remained the only antigen associated with an increased risk of gastric cancer (21).

We previously showed that infection with *H. pylori* might affect immune responses to live oral enteric vaccines, such as *Vibrio cholerae* vaccine CVD 103-HgR (23) and *Salmonella* Typhi vaccine CVD 908-*htrA* (24). Specifically, the immune response was diminished in young Chilean children vaccinated with CVD 103-HgR (23), and enhanced in Malian adults vaccinated with CVD 103-HgR (25), and in US adults vaccinated with CVD 908-*htrA* (24). Given these discrepancies and on the background of the 2013–2014 silent outbreak with WPV1 in Israel during 2013–2014, the aim of the current study was to examine the association of *H. pylori* seroprevalence, and serum pepsinogens (PGs, as markers of gastric inflammation) with the neutralizing antibodies to polio vaccine strains in school-age children.

## Materials and Methods

### Study Design and Population

A seroepidemiological study was conducted among a convenience sample of children aged 5–17 years from northern Israel. Jewish and Arab children were enrolled from Hadera sub-district who attended Hillel Yaffe Medical Center, and from the area of Nazareth city who attended the French Hospital in Nazareth. Children with known immunosuppressive conditions were excluded.

The coverage of OPV vaccination during the 2013–2014 campaign was 79% in the Hadera sub-district and 90% in the northern region of Israel, including Nazareth. Parents of eligible children were interviewed in their native language (Hebrew or Arabic) regarding sociodemographic characteristics and children's health status and medical history.

### Definition of the Study Variables

#### The Dependent Variables

Titers of neutralizing antibody against poliovirus types 1 and 3 vaccine strains were measured using a standard microneutralization assay (26). Children with antibody titer lower than 1:8 were considered unimmunized and unprotected; it is encouraging that only a few children had such low titers. Therefore, we defined the dependent variables, namely high immune response to poliovirus vaccine strains, as having a titer of neutralizing antibodies >1:2,048. This value corresponded to the 60th and 40th percentiles of neutralizing antibody titers against poliovirus type 1 and type 3 vaccine strains, respectively.

#### The Main Independent Variables

The main independent variables were *H. pylori* immunoglobulin G (IgG) seropositivity and serum pepsinogens (PGs) as markers of gastric inflammation (27–29). Children were classified as (1) *H. pylori* positive-CagA positive if they had *H. pylori* IgG antibodies and CagA IgG antibodies; (2) *H. pylori* positive-CagA negative if they had *H. pylori* IgG antibodies, but lacked CagA IgG antibodies; and (3) *H. pylori* negative if they lacked *H. pylori* IgG antibodies. We focused on CagA rather than other antigens, given its strong association with gastric pathology, which is well-established than other *H. pylori* antigens.

An additional classification was based on the combination of *H. pylori* seropositivity with a PGI:PGII ratio ≤6.5 [lowest quartile]. This ratio serves as an indication for severe gastritis, and is similar to clinically relevant mean values in children with gastritis established by endoscopy (27–29). Accordingly, children were grouped as (1) *H. pylori* positive and PGI:PGII ≤6.5 (more severe gastritis); (2) *H. pylori* positive and PGI:PGII>6.5 (without severe gastritis) and (3) *H. pylori* negative if they lacked *H. pylori* IgG antibodies.

#### Covariates

Data were collected on the child's age (in years, continuous variable), sex, population group (Jews or Arabs), maternal age (in years, continuous variable), the number of maternal schooling years (continuous variable), the number of siblings, and the birth order of the child. A household crowding index was calculated by dividing the number of persons living in the household by the number of rooms in the household. These variables might be related to *H. pylori* infection, and were considered as potential confounders.

### Laboratory Methods

Blood samples were obtained from the children centrifuged immediately upon collection, and sera were kept at −20°C until testing. Sera were tested for the presence of *H. pylori* IgG antibodies using commercial ELISA kits (Enzygnost® Anti-*Helicobacter pylori* II/IgG kit, Siemens Diagnostics GmbH, Marburg, Germany) according to the manufacturer's instructions. Sensitivity and specificity values of the kit among children are within the range of 92–97% (30). The detection of *H. pylori* IgG serum antibodies using this kit has been shown to strongly correlate with the detection of *H. pylori* antigen in stool samples using the monoclonal antigen enzyme immunoassay (correlation coefficient = 0.70, *p* < 0.001) (Muhsen K., unpublished). *H. pylori* seropositive sera were tested for IgG antibodies against recombinant CagA protein, using an in-house ELISA protocol (17, 31). Concentrations of serum PGI and PGII were measured using ELISA kits (Biohit Inc., Helsinki, Finland), according to the manufacturer's instructions.

The levels of neutralizing serum antibodies against poliovirus type 1 and 3 were measured at the polio reference laboratory at the Central Virology Laboratory of the Ministry of Health, Israel. The titer of neutralizing antibody against poliovirus type 1 and type 3 vaccine strains was measured in a standard microneutralization assay (26, 32–34), using live attenuated polioviruses (Sabin 1 and Sabin 3).

### Ethical Aspects

The Institutional Review Boards of the Hillel Yaffe Medical Center and the French Hospital, and the ethics committee of Tel Aviv University approved the study protocol. The parents signed a written informed consent.

### Statistical Methods

The study sample was described using means and SD for continuous variables, medians and interquartile range for variables with skewed distribution, and frequencies and percentages for categorical variables.

Sociodemographic differences between children who were *H. pylori* seropositive and negative were examined using the chi square test and Fisher's Exact test as appropriate, and the Student's *t* test for continuous variables and the Mann Whitney test for variables with skewed distribution. Correlations of neutralizing serum antibody titers to poliovirus type 1 and type 3 vaccine strains with the independent variables were assessed using Spearman's correlation coefficient. Differences between children with *H. pylori* seropositivity and negativity, in their having high immune response to poliovirus type 1 and type 3 vaccine strains (see definitions above), were assessed using the chi square test.

To account for potential confounders, we used the inverse propensity score treatment weighting approach (35, 36). We created 3 propensity scores for each main independent variable (35–39). For *H. pylori* seropositivity (positive or negative), a propensity score was created using the predicted probability from the binary logistic regression model. For *H. pylori*/CagA seropositivity (3 categories as outlined above), a propensity score was created using the predicted probability of *H. pylori*/CagA seropositivity from a multinomial logistic regression model with the abovementioned covariates. Inverse probability weights were calculated using the created propensity score by weighting each participant in *H. pylori*/CagA positivity categories (*H. pylori* positive/CagA positive, *H. pylori* positive/CagA negative or *H. pylori* negative) inversely to his/her probability of being classified into these specific categories. A similar approach was followed for the *H. pylori* seropositivity/PGI:PGII ratio. By using the inverse weights, we created pseudo-populations in which *H. pylori* categories were balanced in the distribution of these covariates.

We assessed the association between *H. pylori* seropositivity and high immunity to polioviruses vaccine strains (yes or no) using weighted generalized estimating equations with binary logistic regression, providing a robust variance estimator (39). We also conducted unweighted multivariable logistic regression models that adjusted for confounders using the conventional approach. Similar models were constructed for the main independent variables *H. pylori*/CagA seropositivity and *H. pylori* seropositivity/PGI:PGII ratio. ORs and 95% CIs were obtained from these models.

*P*-value <0.05 was considered statistically significant. The data were analyzed using SPSS software version 25 (IBM, Armonk, New York, USA).

Missing values for the study variables were negligible (<3%); therefore, we performed complete case analysis.

## Results

### The Study Population According to *H. pylori* Seropositivity

Overall, 336 children (63.1% males) were enrolled in the study, of whom 98 (29.2%) were Jewish children and the rest were Arab. The participants' age was in the range of 4.8–17.3 years, with a mean age of 10.7 [standard deviation [SD] 2.3]. Overall, 137 (40.8%) children tested positive for *H. pylori* IgG serum antibody; among these, 51 (37.2%) also tested positive for CagA (virulent phenotype) IgG antibody.

The variables serum pepsinogen (PG) I and PGII levels and the PGI:PGII ratio (measures of gastric inflammation) did not follow normal distributions (*p* < 0.001 by Kolmogorov-Smirnov test). The median of serum PGI and PGII concentration was significantly higher among *H. pylori* seropositive than seronegative children, while the median PGI:PGII ratio was lower among *H. pylori* seropositive children (Supplementary Table 1).

The unweighted analysis showed that *H. pylori* seropositive children were older than seronegative children: 11.1 years and 10.5 years, respectively (*p* = 0.007). The mean number of maternal schooling years was lower among *H. pylori* seropositive than seronegative children: 11.8 and 12.8 years, respectively (*p* < 0.001). The mean crowded index and the mean number of siblings was higher among *H. pylori* seropositive than seronegative children. These differences were balanced in the weighted analysis (Supplementary Table 2). Similar socio-demographic differences were found between the *H. pylori/*CagA positivity groups and the *H. pylori* positivity/PGI:PGII ratio groups (proxy for gastric inflammation); these were balanced in the weighted analyses (Table 1 and Supplementary Table 3).

**Table 1:** The association between sociodemographic variables and *H. pylori*/CagA IgG seropositivity^a^.

	**Unweighted**				**Weighted^b^**			
**Variable**	***H. pylori* positive CagA positive *n* = 51**	***H. pylori* positive CagA negative *n* = 86**	***H. pylori* negative *n* = 199**	***P*-value**	***H. pylori* positive CagA positive *n* = 51**	***H. pylori* positive CagA negative *n* = 86**	***H. pylori* negative *n* = 191**	***P*-value**
Child's age (years), mean (SD)	11.6 (2.3)	10.9 (2.2)	10.5 (2.3)	0.005	10.8 (2.1)	10.6 (2.3)	10.8 (2.5)	0.5
Sex, *n* (%)								
Males	28 (54.9%)	53 (61.6%)	131 (65.8%)	0.3	63.3%	61.2%	61.0%	0.8
Females	23 (45.1%)	33 (38.4%)	68 (34.2%)		36.7%	38.8%	39.0%	
Population group, *n* (%)								
Jewish	13 (25.5%)	21 (24.4%)	64 (32.2%)	0.3	29.6%	25.8%	28.7%	0.5
Arab	38 (74.5%)	65 (75.6%)	135 (67.8%)		70.4%	74.2%	71.3%	
Mean (SD) maternal age, years	39.6 (5.6)	38.3 (5.9)	38.6 (6.2)	0.4	39.0 (5.1)	38.3 (6.0)	38.7 (6.2)	0.3
Mean number of maternal schooling years (SD)	12.2 (2.8)	11.5 (2.7)	12.9 (2.6)	0.001	12.2 (3.2)	12.4 (2.3)	12.5 (2.5)	0.2
Household crowding index, mean (SD)	1.36 (0.49)	1.64 (1.04)	1.29 (0.48)	<0.001	1.37 (0.52)	1.37 (0.71)	1.36 (0.52)	0.8
Birth order, median (IQR)	2 (2)	2 (2)	2 (2)	0.6	2 (2)	2 (2)	2 (2)	0.9
Number of siblings, median (IQR)	3 (2)	3 (2)	2 (1)	0.006	3 (2)	2 (1)	2 (1)	0.18

a *Missing data on maternal age for 1 H. pylori positive CagA negative; on maternal schooling years for 2 H. pylori negatives; household crowding for 5 H. pylori negatives, and on number of siblings for 1 H. pylori negative child*.

b *Propensity score inverse probability weighting for H. pylori/CagA IgG seropositivity*.

*CagA, cytotoxin-associated gene A; IgG, immunoglobulin G; IQR, Interquartile range; SD: standard deviation*.

*P-value by chi square test for categorical variable, ANOVA for continuous variables and Kruskal-Wallis test for discrete variables*.

### Description of the Immune Response Against Poliovirus Types 1 and 3 Vaccine Strains

**Figure 1:**
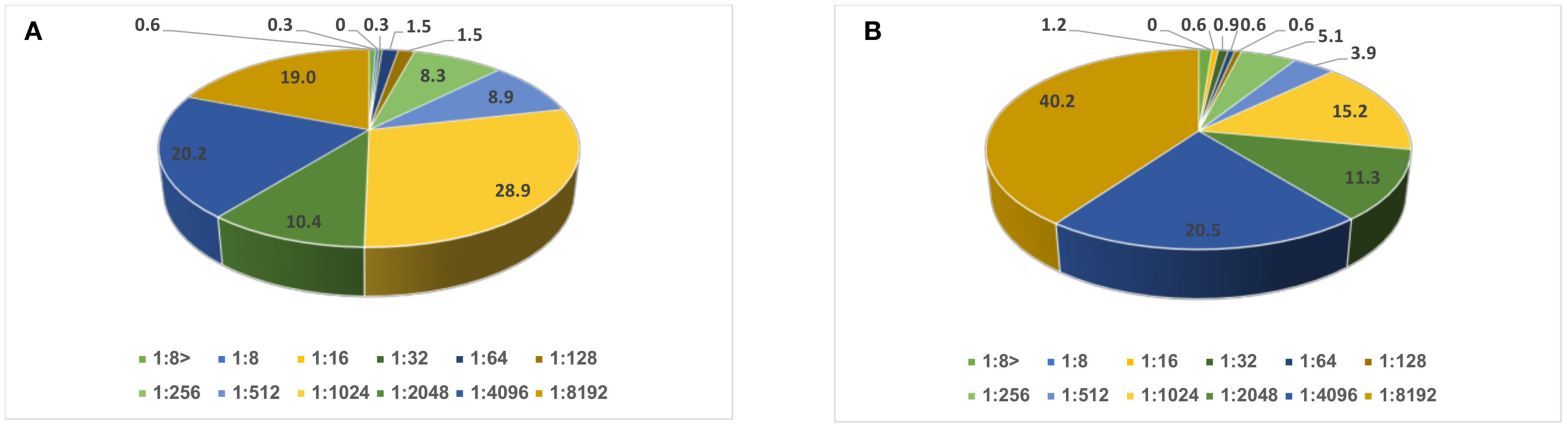
Distribution of neutralizing antibody titer (in percentages): **(A)** against poliovirus type 1 and **(B)** type 3 vaccine strains among school age children (*N* = 336).

A serum neutralizing antibody titer of <1:8 to poliovirus type 1 vaccine strain was found in only 2 (0.6%) children and to poliovirus type 3 vaccine strain in 4 (1.2%) participants. These were considered unimmunized. The remaining participants had a titer ≥1:8 and were considered immunized (Figure 1).

Significant (*p* < 0.001) negative correlations were found between the child's age and neutralizing antibody titers against poliovirus types 1 and 3 vaccine strains (Spearman's correlation coefficients −0.30 and −0.23, respectively). No significant correlations were found between neutralizing antibody titers and other sociodemographic factors (Supplementary Table 4).

### Association of *H. pylori* Seropositivity, Serum Pepsinogens, and Neutralizing Antibody Titer Against Poliovirus Type 1 and 3 Vaccine Strains

In the unweighted analysis, *H. pylori* IgG seropositivity was evident among 46/132 (34.8%) children with high-level immunity to poliovirus type 1 (neutralizing antibody titer >1:2,048) compared to 92/205 (44.9%) among children with neutralizing antibody titer ≤1:2,048, [OR 0.66 (95% CI 0.42–1.03)], but this association was not statistically significant *p* = 0.062. A significant and stronger association was found for the CagA-positive phenotype [OR 0.48 (95% CI 0.25–0.95)], *p* = 0.035, and for *H. pylori* positivity with PGI:PGII ≤6.5 (a proxy of gastric inflammation) [OR 0.41 (95% CI 0.19–0.88)], *p* = 0.021. In the inverse propensity score weighted analysis, these associations were attenuated; but the association with *H. pylori* positivity with PGI:PGII ≤6.5 remained statistically significant [OR 0.43 (95% CI 0.19–0.97)], *p* = 0.041 (Table 2). Associations of *H. pylori* IgG seropositivity, and of *H. pylori* IgG seropositivity according to CagA phenotype and PGI:PGII ≤6.5, with high-level immunity to poliovirus type 3 vaccine strains were of weaker magnitude and mostly non-significant (Table 3). Including the variable “age” in the weighted models slightly strengthened the inverse association between *H. pylori*/PGI:PGII ≤6.5 and high immunity to poliovirus type 1 [OR 0.39 (95% 0.68–0.91), *p* = 0.027]. Each one-year increase in a child's age was associated with a 14%-21% lower odds of having high immunity levels to both poliovirus type 1 and 3 (Table 4). The results were similar in unweighted multivariable logistic regression models that controlled for the child's age, the number of maternal schooling years, household crowding and the number of siblings (Table 5).

**Table 2:** Associations of *H. pylori* IgG seropositivity and serum pepsinogens, with high serum neutralizing antibody level against poliovirus type 1 vaccine strain.

	**Unweighted ^a^**				**Weighted^b^**			
	**High neutralizing serum antibody titer >1:2,048 against poliovirus type 1 (*n* = 132)**	**Neutralizing serum antibody titer ≤1:2,048 against poliovirus type 1 (*n* = 205)**	**OR (95% CI)**	***P*-value^a^**	**High neutralizing serum antibody titer >1:2,048 against poliovirus type 1**	**Neutralizing serum antibody titer ≤1:2,048 against poliovirus type 1**	**OR (95% CI)**	***P*-value^b^**
*H. pylori* seropositivity^b1^				0.062				0.18
Negative	86 (65.2%)	113 (55.1%)	Reference		54.8%	47.0%	Reference	
Positive	46 (34.8%)	92 (44.9%)	0.66 (0.42–1.04)		45.2%	53.0%	0.73 (0.45–1.18)	
By CagA seropositivity^b2^				0.096				0.2
*H. pylori* negative	86 (65.2%)	113 (55.1%)	Reference		38.9%	30.5%	Reference	
*H. pylori* positive CagA negative	32 (24.2%)	54 (26.3%)	0.78 (0.46–1.30)	0.3	34.1%	32.7%	0.82 (0.47–1.43)	0.4
*H. pylori* positive CagA positive	14 (10.6%)	38 (18.5%)	0.48 (0.25–0.95)	0.035	27.0%	36.8%	0.58 (0.28–1.17)	0.12
By PGI:PGII^b3^, ^c^				0.067				0.12
*H. pylori* negative	86 (66.7%)	113 (56.2%)	Reference		39.7%	29.9%	Reference	
*H. pylori* positive, PGI:PGII>6.5	33 (25.6%)	54 (26.9%)	0.96 (0.56–1.64)	0.8	41.8%	42.6%	0.93 (0.54–1.59)	0.7
*H. pylori* positive, PGI:PGII ≤6.5	10 (7.8%)	34 (16.9%)	0.41 (0.19–0.88)	0.021	18.5%	27.5%	0.43 (0.19–0.97)	0.041

a *Odds ratio and 95% confidence intervals and P-values from unweighted logistic regression models*.

b *Propensity score inverse probability weighting for*

b1 *H. pylori seropositivity*

b2 *H. pylori/CagA seropositivity*

b3 *H. pylori seropositivity/PGI:PGII ≤6.5*.

*Odds ratio and 95% confidence intervals and P-values from weighted generalized estimating equations with logistic regression model*.

c *Information on serum pepsinogens was missing for 7 children*.

*CagA, cytotoxin-associated gene A; CI, confidence intervals; IgG, immunoglobulin G; OR, odds ratio; PG, pepsinogen*.

**Table 3:** Associations of *H. pylori* IgG seropositivity and serum pepsinogens, with high serum neutralizing antibody level against poliovirus type 3 vaccine strain.

	**Unweighted^a^**				**Weighted^b^**			
	**High neutralizing serum antibody titer >1:2,048 against poliovirus type 3 (*n* = 204)**	**Neutralizing serum antibody titer ≤1:2,048 against poliovirus type 3 (*n* = 132)**	**OR (95% CI)**	***P*-value^a^**	**High neutralizing serum antibody titer >1:2,048 against poliovirus type 3**	**Neutralizing serum antibody titer ≤1:2,048 against poliovirus type 3**	**OR (95% CI)**	***P*-value^b^**
*H. pylori* seropositivity^b1^				0.064				0.17
Negative	129 (63.2%)	70 (53.0%)	Reference		53.6%	44.5%	Reference	
Positive	75 (36.8%)	62 (47.0%)	0.66 (0.42–1.03)		46.4%	55.5%	0.71 (0.44–1.16)	
By CagA seropositivity^b2^				0.17				0.4
*H. pylori* negative	129 (63.2%)	70 (53.0%)	Reference		36.5%	29.3%	Reference	
*H. pylori* positive CagA negative	47 (23.0%)	39 (29.5%)	0.65 (0.39–1.09)	0.10	32.2%	34.8%	0.71 (0.40–1.25)	0.4
*H. pylori* positive CagA positive	28 (13.7%)	23 (17.4%)	0.66 (0.35–1.23)	0.19	31.3%	35.8%	0.77 (0.39–1.54)	0.2
By PGI:PGII^b3^, ^c^				0.15				0.4
*H. pylori* negative	129 (63.2%)	70 (53.0%)	Reference		35.4%	30.3%	Reference	
*H. pylori* positive, PGI:PGII>6.5	46 (22.9%)	41 (31.8%)	0.78 (0.40–1.53)	0.4	29.9%	38.5%	0.95 (0.46–1.97)	0.8
*H. pylori* positive, PGI:PGII ≤6.5	26 (12.9%)	18 (14.0%)	0.61 (0.37–1.02)	0.057	34.7%	31.1%	0.69 (0.39–1.21)	0.19

a *Odds ratio and 95% confidence intervals and P-values from unweighted logistic regression models*.

b *Propensity score inverse probability weighting for*

b1 *H. pylori seropositivity*

b2 *H. pylori/CagA seropositivity*

b3 *H. pylori seropositivity/PGI:PGII ≤6.5*.

*Odds ratio and 95% confidence intervals and P-values from weighted generalized estimating equations with logistic regression model*.

c *Information on serum pepsinogens was missing for 7 children*.

*CagA, cytotoxin-associated gene A; CI, confidence intervals; IgG, immunoglobulin G; OR, odds ratio; PG, pepsinogen*.

**Table 4:** Age-adjusted propensity score inverse probability weighted models of the association between *H. pylori* seropositivity and high serum neutralizing antibody level (a titer >1:2,048) to poliovirus type 1 and 3 vaccine strains.

	**Polio type 1**		**Polio type 3**	
**Variable**	**Adjusted OR (95% CI)**	***P*-value**	**Adjusted OR (95% CI)**	***P*-value**
*H. pylori* seropositivity				
Negative	Reference		Reference	
Positive	0.72 (0.43–1.18)	0.2	0.69 (0.43–1.12)	0.13
Age, years	0.79 (0.71–0.89)	<0.001	0.85 (0.75–0.95)	0.006
*H. pylori*-CagA seropositivity		0.2		0.3
*H. pylori* negative	Reference		Reference	
*H. pylori* positive CagA negative	0.81 (0.45–1.44)	0.4	0.69 (0.39–1.20)	0.4
*H. pylori* positive CagA positive	0.56 (0.26–1.19)	0.13	0.76 (0.37–1.54)	0.18
Age, years	0.82 (0.72–0.92)	0.001	0.85 (0.74–0.96)	0.012
*H. pylori* seropositivity/PGI:PGII ≤6.5		0.085		0.3
*H. pylori* negative	Reference		Reference	
*H. pylori* positive, PGI:PGII>6.5	0.92 (0.50–1.66)	0.7	0.92 (0.45–1.89)	0.8
*H. pylori* positive, PGI:PGII ≤6.5	0.39 (0.68–0.91)	0.027	0.66 (0.37–1.18)	0.16
Age, years	0.79 (0.68–0.91)	0.002	0.86 (0.75–0.98)	0.023

*CagA, cytotoxin-associated gene A; CI, confidence interval; OR, odds ratio; PG, pepsinogen*.

**Table 5:** Unweighted multivariable logistic regression models of factors associated with high serum neutralizing antibody level (a titer >1:2,048) against poliovirus type 1 and 3 vaccine strains.

	**Polio type 1**		**Polio type 3**	
**Variable**	**Adjusted OR (95% CI)**	***P*-value**	**Adjusted OR (95% CI)**	***P*-value**
Model 1				
*H. pylori* negative	Reference		Reference	
*H. pylori* positive CagA negative	0.81 (0.46–1.42)	0.4	0.67 (0.39–1.17)	0.16
*H. pylori* positive CagA positive	0.60 (0.29–1.21)	0.15	0.76 (0.39–1.45)	0.3
Child's age, years	0.79 (0.71–0.88)	<0.001	0.83 (0.75–0.93)	0.001
Number of maternal schooling years	0.97 (0.88–1.06)	0.4	0.93 (0.85–1.02)	0.13
Household crowding index	0.92 (0.63–1.35)	0.6	0.75 (0.50–1.11)	0.14
Number of siblings	1.02 (0.86–1.20)	0.8	1.02 (0.86–1.19)	0.8
Model 2				
*H. pylori* negative	Reference		Reference	
*H. pylori* positive, PGI:PGII>6.5	0.93 (0.54–1.63)	0.9	0.66 (0.38–1.14)	0.13
*H. pylori* positive, PGI:PGII ≤6.5	0.39 (0.18–0.88)	0.022	0.81 (0.40–1.64)	0.5
Child's age, years	0.79 (0.70–0.88)	<0.001	0.84 (0.75–0.93)	0.001
Number of maternal schooling years	0.97 (0.88–1.06)	0.5	0.92 (0.84–1.01)	0.075
Household crowding index	0.97 (0.67–1.42)	0.8	0.76 (0.51–1.13)	0.17
Number of siblings	0.99 (0.83–1.18)	0.9	0.98 (0.83–1.17)	0.8

*CagA, cytotoxin-associated gene A; CI, confidence interval; OR, odds ratio; PG, pepsinogen*.

In an additional analysis, to minimize the impact of outlier observations we excluded from the analysis 14 children (4.2%) with low neutralizing antibody titers (≤1:128) against poliovirus type 1 vaccine strain, relatively to the rest of the study sample. Neutralizing antibody titer (as a continuous variable) against poliovirus type 1 vaccine strain was significantly lower among *H. pylori* sero-positive participants with evidence of gastric inflammation (PGI:PGII <6.5) compared to *H. pylori* sero-negative children: median levels 1:2,048 vs. 1:1,024, *p* = 0.038 by Mann Whitney U test. No significant difference (*p* = 0.16) was found between the groups in the median levels of neutralizing antibody titer against poliovirus type 3 vaccine strain (the median was 1:4,096 in both groups).

## Discussion

An inverse association was found between *H. pylori* seroprevalence accompanied with gastric inflammation, as evident by the PGI:PGII ratio, and between high neutralizing to poliovirus type 1 vaccine strain among school age children. This association was consistent and robust while using various analytical approaches to account for confounders. The association between overall *H. pylori* sero-prevalence and high immunity to poliovirus type 1 vaccine strain was not significant (p = 0.062).

Overall, the study population had very high immunity levels to polioviruses. This finding is in agreement with previous seroepidemiological studies from Israel (8, 40) and represents the high vaccination coverage (~95%) of poliovirus vaccines in Israel (8).

The observed inverse association between *H. pylori* seroprevalence/PGI:PGII ratio and high immunity to polioviruses corroborates our previous study in Chile. There we reported an inverse association between *H. pylori* sero-prevalence and the immune response to the live attenuated oral cholera vaccine CVD 103-HgR among children aged 6 months to 4 years. However, in older children aged 5–9 years in the same study (23), we found a positive association, as well as in Malian adults (25). Moreover, we found a positive association between *H. pylori* seroprevalence and the humoral immune response to *S*. Typhi vaccine CVD 908-*htrA* in US adults (24). Notably, the immune response to poliovirus in the current study results from vaccination with both intramuscular IPV in early childhood and OPV at school age, during the silent circulation of the wild type poliovirus 1. This limits direct comparison between the observations presented herein and those of previous studies on oral enteric vaccines (23, 24). Collectively, our observations suggest that *H. pylori* seropositivity is related to the immune response to enteric vaccines, regardless of the administration route. Moreover, our findings might have implications to children receiving combined OPV/IPV vaccination schedule, as it is the case in Israel, suggesting that the immune response elicited by combined OPV/IPV vaccines, although in general is high, might be somewhat blunted by *H. pylori*-associated gastric inflammation. The apparently contradictory observations support the notion that the association of *H. pylori* seroprevalence with the immune response to enteric vaccines might vary according to age, i.e., between children and adults. This might be explained by age-related differences in *H. pylori*-induced gastritis. *H. pylori* infection is acquired in early childhood (41), and causes chronic gastritis (42). Although most infected persons remain asymptomatic, the severity of gastric inflammation increases with age, including histopathological changes, even in the absence of symptoms (43). Antrum-predominant-*H. pylori* gastritis, which typically occurs in children, might enhance the secretion of gastric acid. This may explain the inverse association between the infection and an enhanced immune response to poliovirus, since the response is sensitive to acid (44, 45) if the impact is via OPV. Corpus-predominant gastritis causes hypochlorhydria, which usually occurs in adults (42). This might explain the enhanced immune response to the *S*. Typhi and cholera vaccines in adults (24, 25), to acid sensitive pathogens (46, 47).

In the current study, being *H. pylori* sero-positive and having gastric inflammation as measured by PGI:PGII <6.5 was significantly related to high immunity to poliovirus type 1 vaccine strain. However, the association with overall *H. pylori* sero- positivity was not significant (p=0.062), possibly due to limited power. These findings also might suggest that *H. pylori* might plays a role in the immune response to poliovirus vaccine only in a portion of the infected children.

Notably, lower immune response to OPV, measured in terms of seroconversion, in populations from developing countries, was studied in the 1960s and 1970s, especially the immune response to the trivalent OPV (12). This phenomenon was partly attributed to low vaccine uptake due to simultaneous enteric infections at the time of vaccination (11, 14), and the presence of the immunodominant attenuated type 2 poliovirus, which interfered with the response to types 1 and 3 in the vaccine (13). A study conducted by Swartz et al. (14), during 1969–1970 in Tel Aviv metropolitan, showed lower immune response to the trivalent OPV among infants who were vaccinated during the summer than the winter, among infants with vs. without concurrent enteric infections at the time of immunization, and among infants from low vs. high socioeconomic status (14).

*H. pylori* infection affects the microbiome of the stomach and gut (48, 49). A recent study showed a significant effect of enteric infections on the immune response to OPV in children from India, while the gut microbiome did not have any impact in this population (50). In contrast, another study, conducted in the USA, showed that the gut microbiome might affect the immune response to both oral and parental polio vaccines (51, 52). These studies did not assess the role of *H. pylori* infection. The gut microbiome might be a possible link between *H. pylori* infection and the adaptive immune response to vaccines. Among infants, the immune response to IPV might be reduced in relation to maternal poliovirus antibody levels (53). In adults and adolescents, increased age was associated with lower levels of polioviruses sero-positivity in Italy (54). In our study, older children had lower likelihood of having high immunity to poliovirus type 1 and type 3 vaccine strains.

Our study has several strengths. First, we used various markers to characterize *H. pylori* infection, including the measurement of antibodies against CagA (a virulence attribute) and serum PGs as markers of gastric inflammation. Second, we assessed several potential confounders. Third, we utilized various analytical approaches to control for confounders, all of which yielded comparable results. Lastly, the study population sample had near universal immunity to polioviruses. This enabled an exceptional opportunity to assess the relation between chronic infection and attaining the high population level of immunity to polio that is needed to prevent the re-emergence of wild type polioviruses.

Our study has some limitations, including the inability to distinguish between immunity resulting from OPV vs. IPV and between antibodies stimulated by polio vaccines vs. by clinically asymptomatic infection with wild poliovirus. Moreover, the study sample comprised a high proportion of Arab children (70% of the sample), which is not representative of the general population in Israel. However, the sample was representative of the population in the northern region of Israel, where vaccination coverage is higher than in the rest of the country. This, however, is not expected to affect our findings of inverse associations of *H. pylori* seroprevalence plus gastric inflammation and high-level immunity to poliovirus. These associations are generalizable to other populations.

In conclusion, *H. pylori* seroprevalence, accompanied by gastric inflammation, evidenced by a low PGI:PGII ratio, was inversely related to the presence of high titers of serum neutralizing antibody to poliovirus vaccines, in school age children from a population with a high background prevalence of antibodies to poliovirus.

## Data Availability Statement

The datasets presented in this article are not readily available because ethical and legal restrictions apply. Requests to access the datasets should be directed to Khitam Muhsen, kmuhsen@tauex.tau.ac.il.

## Ethics Statement

The studies involving human participants were reviewed and approved by the Institutional Review Boards of the Hillel Yaffe Medical Center and the French Hospital, and the Ethics Committee of Tel Aviv University approved the study protocol. The parents signed a written informed consent. Written informed consent to participate in this study was provided by the participants' legal guardian/next of kin.

## Author Contributions

KM and ML contributed equally to the study as co-PIs and senior authors on this manuscript, planned the study, made substantial contribution to it design, acquired funding, and drafted the manuscript. EK, LB, NE, and MW directed its implementation. EK, LB, NE, KM, and ML contributed substantially to acquisition of data and blood samples. LB and MW were responsible for the laboratory work. KM, LB, and ML were responsible for data analysis. All authors made substantial contributions to interpretation of findings, approved the submitted version of the manuscript, agreed to be personally accountable for the author's own contributions and accuracy of data presented in the manuscript, and contributed to writing and substantive revisions.

## Funding

This study was funded by the United States-Israel Binational Science Foundation (BSF) (PIs KM and ML) grant number 2015361.

## Conflict of Interest

The authors declare that the research was conducted in the absence of any commercial or financial relationships that could be construed as a potential conflict of interest.

## Publisher's Note

All claims expressed in this article are solely those of the authors and do not necessarily represent those of their affiliated organizations, or those of the publisher, the editors and the reviewers. Any product that may be evaluated in this article, or claim that may be made by its manufacturer, is not guaranteed or endorsed by the publisher.
