# Supplementary material for: Relationship Between Helicobacter pylori IgG Seroprevalence and the Immune Response to Poliovirus Vaccine Among School-Age Children From a Population With Near-Universal Immunity Level
Source: Front Med (Lausanne). 2022 Jan 20;8:797719. doi: 10.3389/fmed.2021.797719 (PMC8810818; doi:10.3389/fmed.2021.797719)
Supplement: Supplementary file 1 [file Data_Sheet_1.PDF]

Supplementary Material

Supplementary table 1: Serum pepsinogens levels by *H. pylori* IgG seropositivity

	***H. pylori* positive**	***H. pylori* negative**	**P value [a]**
**Number of samples tested for PGI**	**133**	**193**	
Median PGI μg/L (IQR)	66.0 (36.2)	57.8 (26.7)	<0.001
**Number of samples tested for PGII**	**132**	**193**	
Median PGII μg/L (IQR)	8.6 (5.9)	6.4 (3.6)	<0.001
**Number of samples with data**	**131**	**192**	
Median PGI: PGII (IQR)	7.41 (4.42)	9.34 (4.93)	<0.001

[a] P value by Mann-Whitney test
IgG: Immunoglobulin G; IQR: interquartile range; PG: pepsinogen

Supplementary table 2: The association between sociodemographic variables and *H. pylori* IgG seropositivity

	Unweighted			Weighted [a]		
**Variable**	***H. pylori* positive, n=137**	***H. pylori* negative n=199**	**P value**	***H. pylori* positive**	***H. pylori* negative**	**P value**
**Child's age (years), mean (SD)**	11.1 (2.2)	10.5 (2.5)	0.007	10.7 (2.3)	10.8 (2.5)	0.8
**Sex, n (%)**						
Males	81 (59.1%)	131 (65.8%)	0.2	61.8%	61.8%	1.0
Females	56 (40.9%)	68 (34.2%)		38.2%	38.2%	
**Population group, n (%)**						
Jewish	34 (24.85)	64 (32.25)	0.15	27.0%	28.6%	0.6
Arab	103 (75.2%)	135 (67.8%)		73.0%	71.4%	
**Mean (SD) maternal age, years**	38.8 (5.8)	38.6 (6.2)	0.7	38.6 (5.8)	38.7 (6.2)	0.7
**Mean number of maternal schooling years (SD)**	11.8 (2.8)	12.8 (2.5)	<0.001	12.4 (2.4)	12.5 (2.5)	0.4
**Household crowding index, mean (SD)**	1.54 (0.88)	1.29 (0.48)	0.003	1.37 (0.70)	1.49 (0.51)	0.5
**Birth order, median (IQR)**	2 (2)	2 (2)	0.5	2 (2)	2 (2)	0.6
**Number of siblings, median (IQR)**	3 (2)	2.0 (1)	0.002	2 (1)	2 (1)	0.9

[a] Propensity score inverse probability weighting for *H. pylori* IgG seropositivity
IgG: immunoglobulin G; IQR: interquartile range; SD: standard deviation
P value by chi square test for categorical variable, Student's *t* test for continuous variables and Mann-Whitney test for discrete variables

Supplementary table 3: The association between sociodemographic variables and *H. pylori* seropositivity/PGI:PGII ratio [a]

	Unweighted				Weighted [a]			
Variable	***H. pylori* positive PGI:PGII≤6.5, n=44**	***H. pylori* positive PGI:PGII>6.5, n=87**	***H. pylori* negative n=199**	**P value**	***H. pylori* positive PGI:PGII≤6.5**	***H. pylori* positive PGI:PGII>6.5**	***H. pylori* negative**	**P value**
Child's age (years), mean (SD)	10.9 (2.3)	11.3 (2.3)	10.5 (2.3)	0.021	10.6 (2.2)	10.7 (2.4)	10.8 (2.5)	0.6
Sex, n (%)								
Males	23 (52.3%)	55 (63.2%)	131 (65.8%)	0.2	60.6%	60.6%	61.2%	0.9
Females	21 (47.7%)	32 (36.8%)	68 (34.2%)		39.4%	39.4%	38.8%	
Population group, n (%)								
Jewish	9 (20.5%)	24 (27.6%)	64 (32.2%)	0.2	27.1%	26.5%	28.9%	0.7
Arab	35 (79.5%)	63 (72.4%)	135 (67.8%)		72.9%	73.5%	71.1%	
Mean (SD) maternal age, years	38.5 (5.6)	38.8 (5.9)	38.6 (6.2)	0.9	38.3 (5.4)	38.4 (6.0)	38.6 (6.2)	0.7
Mean number of maternal schooling years (SD)	11.8 (3.3)	11.8 (2.4)	12.9 (2.6)	0.002	12.7 (2.8)	12.4 (2.0)	12.5 (2.5)	0.3
Household crowding index, mean (SD)	1.54 (0.68)	1.55 (0.99)	1.29 (0.48)	0.003	1.40 (0.60)	1.36 (0.75)	1.36 (0.52)	0.5
Birth order, median (IQR)	3 (3)	2 (2)	2 (2)	0.3	2 (2)	2 (2)	2 (2)	0.5
Number of siblings, median (IQR)	3 (2)	3 (2)	2.0 (1)	0.007	2 (2)	3 (1)	2 (1)	0.2

[a] Propensity score inverse probability weighting for *H. pylori* seropositivity/ PGI:PGII ratio
IgG: immunoglobulin G; IQR: interquartile range; PG: pepsinogen; SD: standard deviation
P value by chi square test for categorical variable, ANOVA for continuous variables and Kruskal-Wallis test for discrete variables

Supplementary table 4: Correlations between sociodemographic factors and neutralizing serum antibody against poliovirus type 1 and 3 vaccine strains

Variable	Number	Neutralizing serum antibody titer poliovirus type 1	P value	Neutralizing serum antibody titer poliovirus type 3	P value
Child's age	336	-0.30	<0.001	-0.23	<0.001
Maternal age	335	-0.09	0.08	-0.086	0.11
Maternal schooling years	334	-0.006	0.9	-0.026	0.6
Household crowding index	331	-0.037	0.5	-0.03	0.5
Birth order of the child	336	-0.04	0.4	0,017	0.7
Number of siblings	335	-0.08	0.11	-0.047	0.3

Spearman's correlation coefficient. Neutralizing serum antibody titer was analyzed as a discrete variable
